# Adult mosquitoes of the sibling species *Anopheles gambiae* and *Anopheles coluzzii* exhibit contrasting patterns of susceptibility to four neonicotinoid insecticides along an urban-to-rural gradient in Yaoundé, Cameroon

**DOI:** 10.1186/s12936-024-04876-4

**Published:** 2024-03-02

**Authors:** Fred A. Ashu, Caroline Fouet, Marilene M. Ambadiang, Véronique Penlap-Beng, Colince Kamdem

**Affiliations:** 1 Centre for Research in Infectious Diseases, P.O. Box 13591, Yaoundé 9, Cameroon; 2 Department of Biochemistry, Faculty of Science, University of Yaoundé 1, P.O. Box 11 812, Yaoundé, Cameroon; 3 Department of Biological Sciences, The University of Texas at El Paso, 500 W. University Ave., El Paso, TX 79968 USA

**Keywords:** Insecticide, *Anopheles*, Resistance, Malaria, Neonicotinoids

## Abstract

**Background:**

Neonicotinoids are potential alternatives for controlling pyrethroid-resistant mosquitoes, but their efficacy against malaria vector populations of sub-Saharan Africa has yet to be investigated. The aim of the present study was to test the efficacy of four neonicotinoids against adult populations of the sibling species *Anopheles gambiae* and *Anopheles coluzzii* sampled along an urban-to-rural gradient.

**Methods:**

The lethal toxicity of three active ingredients for adults of two susceptible *Anopheles* strains was assessed using concentration–response assays, and their discriminating concentrations were calculated. The discriminating concentrations were then used to test the susceptibility of *An. gambiae* and *An. coluzzii* mosquitoes collected from urban, suburban and rural areas of Yaoundé, Cameroon, to acetamiprid, imidacloprid, clothianidin and thiamethoxam.

**Results:**

Lethal concentrations of neonicotinoids were relatively high suggesting that this class of insecticides has low toxicity against *Anopheles* mosquitoes. Reduced susceptibility to the four neonicotinoids tested was detected in *An. gambiae* populations collected from rural and suburban areas. By contrast, adults of *An. coluzzii* that occurred in urbanized settings were susceptible to neonicotinoids except acetamiprid for which 80% mortality was obtained within 72 h of insecticide exposure. The cytochrome inhibitor, piperonyl butoxide (PBO), significantly enhanced the activity of clothianidin and acetamiprid against *An. gambiae* mosquitoes.

**Conclusions:**

These findings corroborate susceptibility profiles observed in larvae and highlight a significant variation in tolerance to neonicotinoids between *An. gambiae* and *An. coluzzii* populations from Yaoundé. Further studies are needed to disentangle the role of exposure to agricultural pesticides and of cross-resistance mechanisms in the development of neonicotinoid resistance in some *Anopheles* species.

## Background

The scale-up of vector control has largely contributed to reducing malaria burden over the last two decades in sub-Saharan Africa [1]. Long-lasting insecticidal nets and indoor residual spraying are the cornerstone of malaria prevention and rely on the use of eight classes of chemical insecticides [2]. Prior to the recent prequalification of formulations based on new active ingredients (i.e., chlorfenapyr, clothianidin, etofenprox and broflanilide) by the World Health Organization (WHO), neurotoxic insecticides that disrupt a sodium channel or inhibit acetylcholinesterase in the insect’s nervous system were widely applied [3]. The similarity of modes of action and the intensive use of a limited number of active ingredients have created ideal conditions for the emergence and spread of resistance [4, 5]. Indeed, resistance to any of the classes of neurotoxic insecticides used in interventions has been reported posing a challenge to the sustainability of vector control [6, 7]. As a result, the search for new insecticides has become an urgent necessity [8, 9]. In the quest for new active ingredients, alternatives to sodium channel and acetylcholinesterase inhibitors have drawn considerable attention because their new modes of action are more suitable for targeting populations that are currently resistant to existing insecticides [10–12].

Formulations of two neonicotinoid insecticides repurposed from the agricultural sector (clothianidin and imidacloprid) are prequalified for indoor residual spraying (IRS) and space spraying targeting malaria-carrying mosquitoes [2]. Clothianidin is an active ingredient used alone or in combination with deltamethrin in four new IRS formulations [2, 13–15]. Imidacloprid is combined with prallethrin in Cielo ULV®, a formulation manufactured for space spraying. Neonicotinoids act as agonist of acetylcholine, selectively target the insect nicotinic acetylcholine receptor (nAChR) and disrupt excitatory cholinergic neurotransmission leading to paralysis and death [16].

Cross-resistance which occurs when insects develop resistance to several active ingredients that have a similar mode of action is a pervasive driver of tolerance to neonicotinoids in agricultural pests [17–20]. Importantly, significant research has demonstrated that detoxification enzymes, primarily cytochrome monooxygenases (CYPs), play a key role in the development of neonicotinoid resistance in insect pests [21–25]. Due to chronic exposure to public health and agricultural pesticides and other xenobiotic compounds, anopheline populations from sub-Saharan Africa have evolved an impressive array of detoxification mechanisms including overexpression and duplication of CYPs [26–30]. These enzymes represent a threat to the efficacy of neonicotinoids as vector populations can use preexisting metabolic and excretion mechanisms selected by previous exposure to adapt to new active ingredients.

Agricultural spray of pesticides is also known to be a major factor driving resistance to public health insecticides [31, 32]. Neonicotinoids are intensively used in agriculture and represented more than 25% of the global insecticide sales share in 2014 [33]. In some sub-Saharan African countries, between 100 and 200 formulations of thiacloprid, imidacloprid, acetamiprid and thiamethoxam are registered for agricultural pest management [34]. Neonicotinoids sprayed to protect crops from insect pests are highly water-soluble and are prone to leach in aquatic habitats that are also used as breeding sites by *Anopheles* larvae in farming areas [35, 36]. This unintentional exposure may contribute to the development of cross-resistance to neonicotinoids in larval populations [37–40].

To better evaluate the risk of cross-resistance to neonicotinoids in *Anopheles* mosquitoes, it is vital to assess baseline susceptibility of vector populations to a wide range of neonicotinoid insecticides. Notably, addressing the fine-scale variations in susceptibility between species and geographic areas could provide insights into the candidate environmental and genetic drivers of resistance. Larvae of the sibling species *Anopheles gambiae* and *Anopheles coluzzii* from urban and suburban areas of Yaoundé display contrasting patterns of resistance to neonicotinoids [38]. In contrast to *An. coluzzii*, third instar larvae of *An. gambiae* can growth and emerge in water containing a concentration of neonicotinoid that kill immature stages of a susceptible strain within 24 h [39]. Therefore, comparing patterns of resistance and susceptibility between populations of the two sibling species could help decipher the key players involved in the development of neonicotinoid resistance in anopheline populations.

The present study aimed to test the efficacy of four agricultural neonicotinoids against *An. gambiae* and *An. coluzzii* adult mosquitoes along an urban-to-rural gradient in Yaoundé, Cameroon. The lethal toxicity of three active ingredients was tested, and the susceptibility of wild female adults to clothianidin, acetamiprid, imidacloprid and thiamethoxam assessed. *Anopheles coluzzii* populations that thrive in urban areas of Yaoundé were globally susceptible to neonicotinoids, while *An. gambiae* adults collected from rural and sub-urban environments were more tolerant. The potential efficacy of neonicotinoid formulations against malaria mosquitoes could be impacted by fine-scale geographic variations in susceptibility among wild populations as well as the low toxicity of some active ingredients.

## Methods

### Lethal concentrations determination

Center for Disease Control and prevention (CDC) bottle bioassays were used to assess the lethal toxicity of neonicotinoids [41]. Four active ingredients were tested: acetamiprid, imidacloprid, thiamethoxam and clothianidin. Acetamiprid, imidacloprid and thiamethoxam are commonly used by farmers in Cameroon to protect several types of crops from insect pests [34, 42]. Clothianidin is an agrochemical, which is not registered in Cameroon, but whose formulations have been approved for malaria mosquito control [2]. All four neonicotinoids tested were technical-grade material (Sigma Aldrich, Pestanal®). The insecticides were dissolved in absolute ethanol except imidacloprid for which acetone was used. A range of concentrations of the active ingredient was tested against a susceptible strain (*An. gambiae* Kisumu or *An. coluzzii* Ngousso) to determine LC_50_ and LC_99_ corresponding to the lowest concentrations required to kill 50% and 99% of susceptible individuals, respectively. By contrast to clothianidin whose toxicity has been tested with at least one susceptible strain [8, 12, 42, 43], information on the lethal concentrations of acetamiprid, imidacloprid and thiamethoxam against African malaria mosquitoes is lacking. The three active ingredients were tested using the following gradients: imidacloprid (12.5, 50, 100, 200 and 250 µg/ml); acetamiprid (12.5, 25, 50, 75 and 150 µg/ml); thiamethoxam (3, 50, 150, 250 and 300 µg/ml).

250-ml Wheaton bottles were coated with 1 ml of a given concentration of the insecticide and 25 female adult mosquitoes, 3 to 5 days old, were exposed for 1 h in the bottles. After the exposure period, mosquitoes were removed from the bottles and released into net-covered paper cups on top of which cotton imbibed with 10% sugar solution was placed. Mortality was observed at 24 h and 72 h, respectively. Bioassays were performed under a controlled environment of 25–27 °C, 70–90% relative humidity and a 12:12 h light/dark photoperiod. Four replicates were tested per concentration together with two controls where mosquitoes were exposed to 1 ml of solvent, ethanol or acetone.

### Susceptibility evaluation in wild populations

**Fig. 1:**
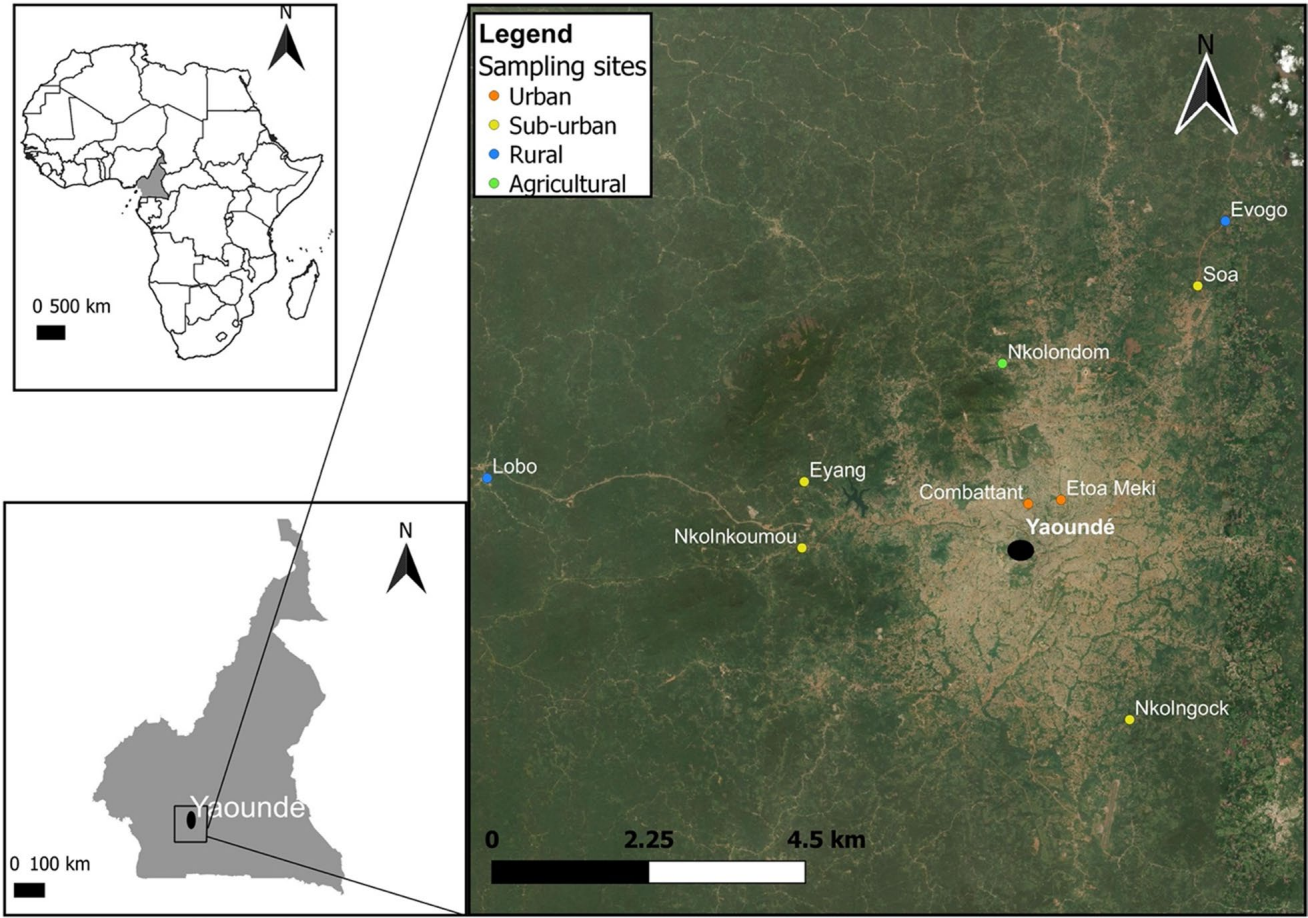
Map of the sampling sites where neonicotinoid susceptibility was monitored in *An. gambiae* and *An. coluzzii* adults. The nine sites are located along an urban-to-rural gradient centered on Yaoundé. The city (brown areas) situated in the equatorial zone of Cameroon is surrounded by degraded forests (green areas)

Wild *An. gambiae* sensu lato (s.l.) (the *An. gambiae* complex) mosquito populations were collected from several field surveys and tested between September 2019 and September 2022. Mosquito larvae were sampled using a dipper from breeding sites identified in nine locations along an urban-to-rural gradient in Yaoundé. Two locations surveyed within the city were densely urbanized (Combattant, Etoa Meki), while seven sampling sites belonged to suburban and rural areas (Fig. 1). One of the suburban sites (Nkolondom) was situated in a neighbourhood where marshy areas are being used for intensive cultivation of food crops (Fig. 1) [37].

Larvae collected from typical *An. gambiae* s.l. breeding sites were transported in plastic containers to the insectary where they were identified using reference morphological identification keys [44, 45]. Larvae were reared in trays containing 200 ml borehole water and fed with TetraMin® daily. Adults that emerged were maintained in 30 cm-by-30 cm cages and provided with 10% sugar solution. Previous studies have established that two cryptic species of the *An. gambiae* complex: *An. gambiae *sensu stricto (hereafter referred to as *An. gambiae*) and *An. coluzzii* segregate along an urbanization gradient in Yaoundé [46, 47]. *Anopheles coluzzii* is found exclusively in densely urbanized settings, while *An. gambiae* occurs in sub-urban and rural environments. The two species were identified by subjecting a subset of 50 female adults from each sampling site to a molecular diagnostic method described in [48]. Female adults were tested to assess their level of susceptibility to neonicotinoids using bottle bioassays and a discriminating concentration of the insecticide. The discriminating concentration, defined as the lowest concentration required to kill 100% from a susceptible strain (*An. gambiae* Kisumu or *An. coluzzii* Ngousso), was chosen within the confidence intervals of LC_99_ for acetamiprid, imidacloprid and thiamethoxam. The discriminating concentration of clothianidin was obtained from Agumba et al*.* [42].

### Synergistic effect of piperonyl butoxide

A bioassay was performed to test if the synergistic effect of the cytochrome P450 inhibitor, piperonyl butoxide (PBO) could restore neonicotinoid susceptibility. Female adults that emerged from the same pool of larvae were first exposed to 4% PBO in CDC bottles, for 1 h before being released into other bottles coated with the neonicotinoid insecticide. Susceptibility tests were conducted as described above. Mortality values obtained with or without prior exposure to the synergist were compared after 72 h of holding period.

### Data analysis

The mortality rate was calculated as a percentage across all four test bottles. Abbott’s formula was used to correct mortality rates when 5–20% of individuals died in the corresponding control tests [49]. For each insecticide, a log-logistic model were used to fit the concentration–response curve with the *drc* package in R (version 4.2.2) [50]. A probit model was applied to determine LC_50_ and LC_99_ and their 95% confidence intervals for each insecticide using the *ecotox* package. To compare LC_50_ values at 24 and 72 h, a ratio test implemented in Wheeler et al*.* [51] was used. Mortality in wild populations was interpreted based on the WHO criteria which suggest that 98%–100% mortality indicates susceptibility, 90%–97% mortality reflects the possibility of resistance that needs to be confirmed and less than 90% mortality corresponds to resistance [52].

## Results

### Lethal concentrations of three neonicotinoids against *An. gambiae* and *An. coluzzii*

**Fig. 2:**
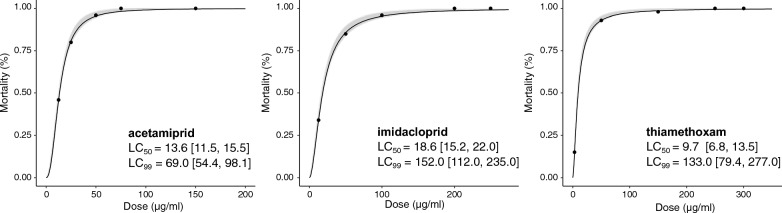
Concentration–response curves with the standard error of the regression model (grey bands) depicting the 24-h toxicity of three neonicotinoids against *Anopheles* mosquitoes. LC_50_ and LC_99_ with 95% confidence intervals of acetamiprid and imidacloprid were determined using the susceptible strain *An. gambiae* Kisumu while lethal concentrations of thiamethoxam were estimated with the *An. coluzzii* Ngousso strain

The acute toxicity of three neonicotinoids against *Anopheles* mosquitoes was tested using concentration–response bioassays with mortality recorded within 24 h and 72 h of exposure. Results of LC_50_ and LC_99_ of acetamiprid, thiamethoxam and imidacloprid are presented in Table 1 and Fig. 2. 24-h LC_99_ obtained with the susceptible strain *An. gambiae* Kisumu were 69.0 µg/ml, confidence interval CI_95%_ (54.40, 98.1), for acetamiprid compared to 152.0 µg/ml (112.0, 235.1) for imidacloprid. Meanwhile, 24-h lethal concentrations of thiamethoxam detected using the susceptible strain *An. coluzzii* Ngousso were 9.6 µg/ml (6.8, 13.5) for LC_50_ and 133.0 µg/ml (79.4, 277.0) for LC_99_, respectively. Low lethal concentrations reflect high toxicity since smaller doses of insecticide would be required to kill a given number of exposed individuals. Based on the lowest LC_50_, thiamethoxam [LC_50_: 9.6 µg/ml (6.8, 13.5)] was the most toxic neonicotinoid to *Anopheles* mosquitoes within 24 h followed by acetamiprid [LC_50_: 13.6 µg/ml (11.5, 15.5)] and imidacloprid [LC_50_: 18.6 µg/ml (15.2, 20.0)]. To compare lethal toxicity within 24 h and 72 h, a ratio test was applied. The toxicity was considered different between 24 and 72 h if there was a significant difference between LC_50_ values. Based on the ratio test, the difference in LC_50_ at 24 h and 72 h for the three active ingredients tested was not significant (*p* > 0.05), which suggested that extending the holding period did not increase toxicity (Table 1).

**Table 1:** Lethal concentrations, LC_50_ and LC_99_, estimated at 24 h and 72 h post-exposure

Active ingredient	Species	Number of mosquitoes	Holding period (h)	LC_50_ value (µg/L)	Lower–upper 95% confidence intervals (µg/L)	Standard error	LC_99_ value (µg/L)	Lower–upper 95% confidence intervals (µg/L)	Standard error	Chi square	*p* value
Acetamiprid	*An. gambiae* (Kisumu)	500	24	13.60	11.50–15.50	1.08	69.00	54.40–98.10	1.16	18.10	< 0.001
			72	13.90	11.30–16.20	1.07	64.10	48.70–101.00	1.15	24.30	< 0.001
Imidacloprid	*An. gambiae* (Kisumu)	350	24	18.60	15.20–22.00	1.10	152.00	112.00–235.00	1.20	18.40	< 0.001
			72	17.10	12.90–21.40	1.10	148.00	100.00–273.00	1.21	22.00	< 0.001
Thiamethoxam	*An. coluzzii* (Ngousso)	475	24	9.66	6.78–13.50	1.14	133.00	79.40–277.00	1.26	25.70	< 0.001
			72	7.47	5.84–9.67	1.13	75.30	47.8–142.00	1.31	13.20	< 0.001

### Variation in susceptibility within and between species

The baseline susceptibility of wild populations to acetamiprid, imidacloprid, and thiamethoxam, was evaluated using discriminating concentrations of 75 µg/ml, 200 µg/ml and 250 µg/ml, respectively. The concentrations were chosen within the 95% confidence intervals for 24-h LC_99_ with the aim to balance the risk of not detecting low-level resistance while limiting the risk of reporting false positives. In addition to acetamiprid, imidacloprid and thiamethoxam, we tested a fourth neonicotinoid (clothianidin) using a discriminating concentration of 150 µg/ml. The efficacy of the discriminating concentrations against the insecticide susceptible strains *An. gambiae* Kisumu and *An. coluzzii* Ngousso was confirmed. For both laboratory strains, 100% of female adults exposed to the discriminating concentration of each of the four neonicotinoids died within 24 h.

**Fig. 3:**
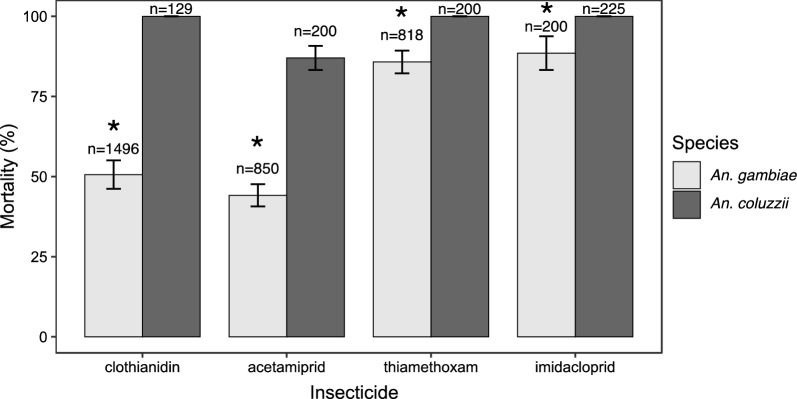
Percent mortality in *An. gambiae* and *An. coluzzii* female adults exposed to four neonicotinoids. Error bars indicate the standard error of the mean and (n) the total number of adult mosquitoes tested from several pools of larvae collected between 2019 and 2022. *Fisher’s exact test indicated a significantly lower mortality in *An. gambiae* compared to *An. coluzzii* (*p* < 0.05)

Molecular analyses confirmed that *An. gambiae* s.l. larvae collected from two sites situated in urbanized areas (Etoa Meki and Combattant) were 100% *An. coluzzii*. Across two sites located in the sub-urban area (Nkolkoumou and Soa), the relative frequencies of *An. gambiae* and *An. coluzzii* were ~ 80% and 20%, respectively. *Anopheles gambiae* was the only member of the species complex collected from the remaining five sites. *Anopheles coluzzii* mosquitoes that occurred in urban areas were susceptible to neonicotinoids except acetamiprid for which signs of reduced susceptibility were apparent (Fig. 3). 100% mortality was observed within 72 h of exposure to thiamethoxam, imidacloprid and clothianidin whereas the average 72-h mortality for acetamiprid was 80%. Mosquito populations from Nkolnkoumou were susceptible to imidacloprid and clothianidin and resistant to thiamethoxam and acetamiprid. Samples collected from Soa were tested only with clothianidin and were susceptible. None of the populations from typical *An. gambiae* habitats (rural/sub-urban areas) were susceptible to acetamiprid or clothianidin. Populations from the agricultural site (Nkolondom) were the least susceptible to neonicotinoids, with mortality rates lower than 50% for acetamiprid and clothianidin. It was also the only site where resistance to all four neonicotinoids was detected.

Using Fisher’s exact test, the susceptibility profiles of wild populations of *An. gambiae* (n = 3364) and *An. coluzzii* (n = 754) to the four neonicotinoids was compared. Mortality rates within 72 h of insecticide exposure was significantly lower in *An. gambiae* compared to *An. coluzzii* for acetamiprid (*p* < 2.2E−16), clothianidin (*p* < 2.2E−16), imidacloprid (*p* = 1.46E−08) and thiamethoxam (*p* = 1.86E−12) (Fig. 3).

**Fig. 4:**
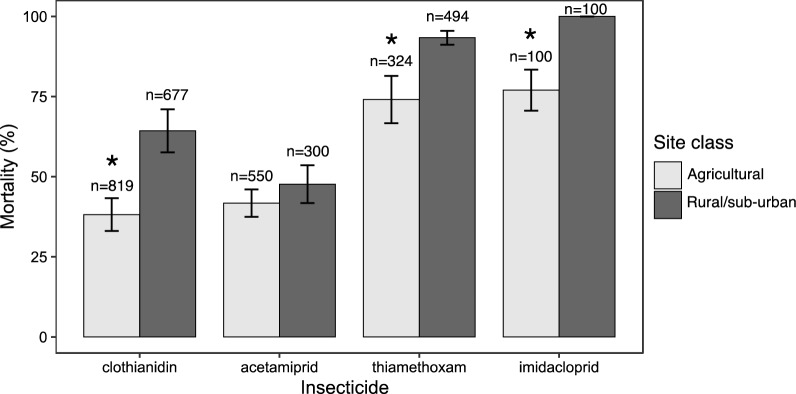
Mortality rates in *An. gambiae* female adults exposed to a discriminating concentration of a neonicotinoid. n = sample size, *Fisher’s exact test indicated a significantly lower mortality in the agricultural site compared to rural/sub-urban populations (*p* < 0.05)

The susceptibility profiles of populations from the agricultural site (Nkolondom, n = 1793) and those from the rural and sub-urban areas (Soa, Nkolnkoumou, Evogo, Nkolngock, Eyang and Lobo, n = 1571) revealed significant differences. Female adults from Nkolondom exhibited significantly lower mortality compared to rural/sub-urban populations for clothianidin (*p* < 2.2E−16, Fisher’s exact test), imidacloprid (*p* = 5.71E−08) and thiamethoxam (*p* = 3.12E−14), but not for acetamiprid (*p* = 0.1156) (Fig. 4). While thiamethoxam was the most toxic to *Anopheles* mosquitoes based on the lowest LC_50_, imidacloprid was the most effective considering the mortality rate induced in wild populations. Clothianidin was the least effective in *An. gambiae* populations from rural/sub-urban and agricultural areas.

### PBO enhances neonicotinoid activity in *An. gambiae*

**Fig. 5:**
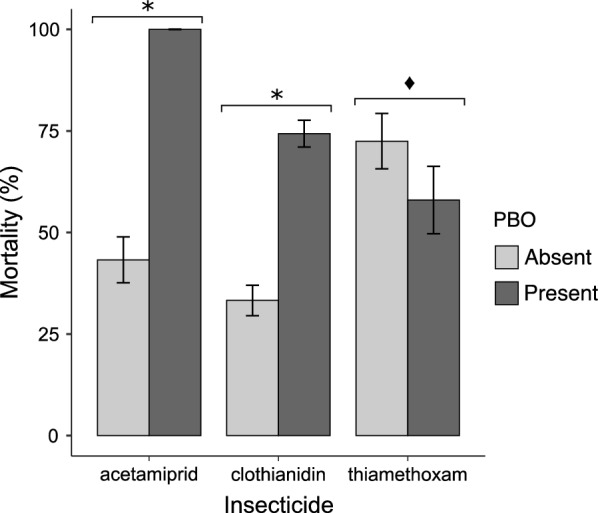
Effects of piperonyl butoxide (PBO) on the efficacy of three neonicotinoids against resistant *An. gambiae* mosquitoes. *Mortality was significantly higher in the presence of PBO (*p* < 0.05, Fisher’s exact test). Black diamond suit: mortality was significantly lower in the presence of PBO (*p* < 0.05)

Bioassays were carried out to determine if inhibition of cytochromes mediated by PBO could improve the efficacy of neonicotinoids against *Anopheles* mosquitoes. The synergistic effect of PBO was tested using the agricultural population that was resistant to the four neonicotinoids. Clothianidin, acetamiprid and thiamethoxam were tested because mortality rates within 72 h of exposure were less than 75% allowing a more accurate evaluation of synergism. Susceptibility was fully restored for acetamiprid (100% vs 35.55 ± 5.25%, *p* < 2.2E−16, Fisher’s exact test) and partially restored for clothianidin (74.33 ± 3.82 vs 30.98 ± 3.49, *p* < 2.2E−16) in the presence of PBO at 72 h post-exposure (Fig. 5). On the contrary, pre-exposure to PBO reduced mortality to thiamethoxam (58.0% ± 8.2 vs 71.5% ± 7.7,* p* = 0.04149, Fisher’s exact test).

## Discussion

Most insecticides used in mosquito control come from the agricultural sector [5]. A new generation of active ingredients are being evaluated to control anopheline mosquitoes that have developed resistance to existing vector control insecticides [8, 9]. In the present study, neonicotinoids, a class of insecticides including clothianidin and imidacloprid whose formulations are proposed for indoor residual spraying and space spraying were tested [13–15].

The lethal concentrations of three different neonicotinoids were determined using two susceptible laboratory strains. 24-h lethal concentration indicated that thiamethoxam was the most toxic neonicotinoid to anopheline mosquitoes as it had the lowest LC_50_, followed by acetamiprid [42]. However, the neonicotinoids tested relatively low toxicity against *Anopheles* mosquitoes based on LC values. For example, in comparison with the pyrethroid deltamethrin, the 24-h LC_99_ of the most potent neonicotinoid was approximately sevenfold lower [41] A large-scale screening conducted to search for candidate pesticides that could be used for malaria vector control revealed that three neonicotinoids were among the least active insecticides against adults of *Aedes aegypti* and *Anopheles stephensi* in a list of nearly 100 compounds tested [9]. LC_80_ of > 200 µg/ml was obtained in 24 h when an insecticide-susceptible strain of *An. stephensi* was exposed to imidacloprid or thiamethoxam while LC_80_ for acetamiprid was ~ 20 µg/ml [9]. In the current study, it was also noted that extending the holding period post-exposure did not improve the toxicity of neonicotinoids as there was no significant difference between LC_50_ at 24 h and 72 h. Two laboratory strains (*An. gambiae* Kisumu and *An. coluzzii* Ngousso) were used to determine the discrimination concentrations that were applied to evaluate susceptibility in wild populations. Although the values detected provided sufficient discriminating power, a more comprehensive survey involving multiple laboratory colonies is still needed to establish guidelines on neonicotinoid susceptibility testing in malaria vectors [43, 53].

This study revealed a reduction in susceptibility to neonicotinoids in wild anopheline mosquito populations. Contrasting patterns of baseline susceptibility to neonicotinoids between the two sibling species *An. gambiae* and *An. coluzzii* were observed. These results corroborated findings from earlier surveys indicating that clothianidin resistance is emerging in *An. gambiae* [38]. In contrast to clothianidin which is not registered for crop protection, formulations of acetamiprid and imidacloprid are intensively used in agricultural areas in some African countries including Cameroon [34, 38, 54]. Agricultural pesticides have historically play a key role in the spread of resistance to insecticides used in public health programmes among malaria-carrying mosquito species [31, 55]. As neonicotinoids are highly soluble in water and are likely to leach in aquatic habitats, it has been suggested that exposure of larval populations to pesticide residues may contribute to resistance selection [35, 55]. Indeed, a complimentary study has revealed that *An. gambiae* larvae collected from rural and suburban areas of Yaoundé displayed high fitness when reared in water containing concentrations of neonicotinoids that were lethal to susceptible strains [38]. For instance, at least 40% of larvae collected from Nkolondom and Nkolnkoumou were able to survive and emerge in water containing a lethal concentration of acetamiprid or imidacloprid [39]. Although the environmental and genetic bases of neonicotinoid tolerance in female adult mosquitoes have yet to be investigated, the present study suggests that selection at larval stage could be a driver of cross-resistance to several active ingredients [38–40]. *Anopheles coluzzii* populations collected from urbanized areas of Yaoundé had sub-optimal mortality (~ 80%) to acetamiprid, but this species was generally susceptible to neonicotinoids. This finding is also consistent with larval tests, which showed that third instars of *An. coluzzii* from Yaoundé had low survival and barely emerged in water containing a lethal concentration of a neonicotinoid [38]. However, in Ivory Coast, reduced susceptibility to acetamiprid and imidacloprid has been reported in adults of *An. coluzzii* sampled from agricultural areas suggesting that populations of this species can develop resistance to neonicotinoid [37].

Although our data show that neonicotinoid resistance was stronger among populations collected from an agricultural site, it is premature to establish a link between residual pesticide exposure in farming areas and the development of resistance. The role of cross-resistance mechanisms notably the contribution of agricultural pesticides and of detoxification enzymes on resistance selection has yet to be elucidated. In this study, it was noted that pre-exposure of resistant *Anopheles* mosquitoes to PBO restored their susceptibility to acetamiprid suggesting that cytochrome P450 monooxygenases (CYPs) are involved in resistance to this insecticide. Indeed, overexpression of CYPs is an important mechanism underlying neonicotinoid resistance in a variety of insect pests including mosquitoes, white fly and aphids [22, 38, 56].

Gene expression analysis revealed overexpression of multiple CYP genes in an acetamiprid-resistant strain of the melon aphid, *Aphis gossypii*, indicating a role of P450-mediated detoxification in acetamiprid resistance [57]. Acetamiprid resistance has also been shown to depend strongly on monooxygenases in white flies and *Aedes* mosquitoes [23, 24]. Consistent with past surveys, the current study confirms that pre-exposure to PBO also drastically improve the efficacy of clothianidin against *Anopheles* mosquitoes [38]. Conversely, PBO had a slightly negative effect on the activity of thiamethoxam in wild populations. This could be explained by the fact that thiamethoxam is a pro-insecticide which needs to be converted to clothianidin before being active in insects. This conversion is catalyzed by enzymes which might have been inhibited by PBO, leading to a slight reduction in insecticidal activity when mosquitoes were pre-exposed to PBO [58].

Some surfactants have been shown to enhance the efficacy of neonicotinoids against malaria vectors [8, 37, 40, 59]. This synergistic action could be harnessed to improve neonicotinoid formulations that may be used vector control. In the present study, it has been observed that PBO is a synergist of neonicotinoids, which offers additional options to enhance the potency of some active ingredients against *Anopheles* mosquitoes.

The contrasting pattern of susceptibility observed between *An. gambiae* and *An. coluzzii* from Yaoundé highlights the need for a more comprehensive survey of tolerance to neonicotinoids in malaria vectors species [60]. The vectorial system in sub-Saharan Africa comprises at least a dozen major species that occupy diverse niches at larval and adult stages [61, 62]. The results show that the susceptibility profile could vary significantly within and between *Anopheles* species, even on a small geographic scale. Species including *An. gambiae*, *An. coluzzii* and *Anopheles arabiensis* whose larvae are more likely to be exposed to neonicotinoid residues in man-made habitats such as temporary breeding sites created in farms should be the focus of extensive monitoring efforts [63, 64]. Even if *Anopheles funestus* larval habitats are less prone to anthropogenic disturbance, populations of this species have developed strong pesticide resistance based on overexpression of detoxification enzymes [27, 65, 66]. Preselected enzymes can contribute to the detoxification of some neonicotinoid insecticides and to the emergence of resistance in *An. funestus* [66].

## Conclusions

Although neonicotinoids have low acute toxicity and reduced efficacy in some *Anopheles* mosquito populations, the use of more potent formulations can still provide alternatives for controlling pyrethroid-resistant malaria mosquitoes. The current study and complimentary investigations revealed that synergists and adjuvants such as PBO and surfactants could be used to enhance the efficacy of neonicotinoid insecticides against *Anopheles* mosquitoes.

## Data Availability

The data for this study have been presented within this article.
